# Human primary skeletal muscle cells express glutamate receptor GluR3, are activated by glutamate, and are affected by autoimmune GluR3B antibodies of epilepsy patients

**DOI:** 10.3389/fphys.2025.1636766

**Published:** 2025-12-11

**Authors:** Mia Levite, Nili Ilouz, Avi Harazi, Hadassa Goldberg-Stern, Eithan Galun, Stella Mitrani-Rosenbaum

**Affiliations:** 1 Faculty of Medicine, The Hebrew University, Jerusalem, Israel; 2 Institute of Gene Therapy, Hadassah Hebrew University Medical Center, Jerusalem, Israel; 3 Sackler Faculty of Medicine, Tel Aviv University, Tel Aviv, Israel; 4 Institute of Pediatric Neurology, Epilepsy Center, Schneider Children’s Medical Center, Petah Tiqva, Israel

**Keywords:** human skeletal muscle, glutamate, glutamate receptor, GluR3, GluR3 **B** antibodies, epilepsy, autoimmune epilepsy, nodding syndrome

## Abstract

**Background:**

Glutamate is the major excitatory neurotransmitter in the nervous system, common in neuromuscular junctions, and with abnormally reduced levels in several muscle diseases. Glutamate receptor AMPA GluR3, encoded by the GRIA3 gene, has important neurophysiological roles in regulation of neural networks, sleep, and breathing. GluR3 deletion or abnormal function increases the susceptibility to seizures and disrupts oscillatory networks of sleep, breathing, exploratory activity, and motor coordination.

**Questions:**

Do human skeletal muscle cells express GluR3? Are they activated by glutamate? Do autoimmune GluR3 **B** antibodies of Nodding Syndrome (NS) patients, and/or other intractable epilepsy patients, that bind and damage neural cells, also bind and affect skeletal muscle cells?

**Results:**

We discovered several original findings: 1) Human primary skeletal muscle cells (myoblasts) express GluR3 RNA and protein, evident by PCR and immunostaining, 2) glutamate (10^−8^–10^−5^M) increases intracellular sodium in human skeletal muscle cells and increases muscle cell number (probably by inducing muscle cell proliferation), 3) AMPA and NMDA increase intracellular sodium in skeletal muscle cells, 4) GluR3 **B** monoclonal antibody binds skeletal muscle cells and increases their number, 5) autoimmune affinity-purified GluR3 **B** antibodies of epileptic NS patients, suffering from nodding due to loss of muscle tone and muscle wasting, bind skeletal muscle cells, 6) purified IgGs rich in autoimmune GluR3 **B** antibodies of intractable epilepsy patients bind and kill skeletal muscle cells.

**Possible implications:**

Together, the novel findings in this study may have various important implications on muscle physiology and pathology and call for continuation studies on diverse physiological, pathological and therapeutic topics. Meanwhile, we raise few hypotheses: 1) GluR3 has an important physiological role in muscle cells and motor function, 2) impaired GluR3 function (due to genetic/epigenetic/autoimmune/infectious/inflammatory factors?) can cause muscle impairments and motor problems, 3) glutamate, by direct activation of GluR3 and/or other GluRs expressed in skeletal muscle cells, can beneficially affect muscle cell survival, growth, and function, 3) Glutamate, iGluR agonists, and/or GluR3 **B** mAb may have therapeutic effects for muscle diseases, injuries, and age-related sarcopenia, 4) autoimmune GluR3 **B** antibodies of NS patients and/or other epilepsy patients may bind GluR3 in muscle cells, damage these cells, and induce muscle dysfunction and motor problems.

## Graphical Abstract

**Figure:**
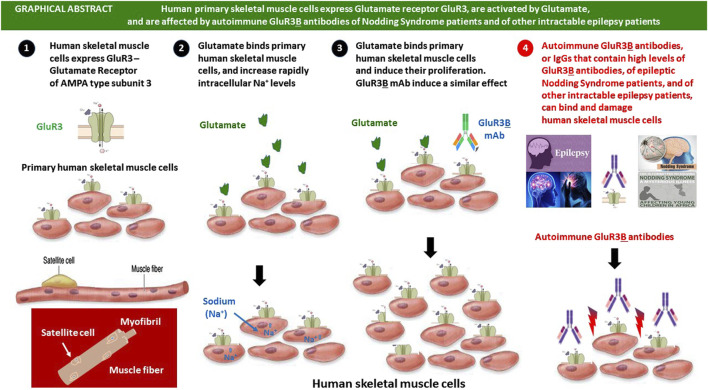

## 1 Introduction

### 1.1 Glutamate in the nervous system

Glutamate is the most abundant, potent, and important excitatory neurotransmitter in the mammalian central nervous system (CNS). Glutamate is released primarily by nerve cells in the brain, activates glutamate receptors of multiple types (Reiner and Levitz, 2018; Hansen et al., 2021), and induces multiple effects critical for intact neural activities and essential brain functions, including cognition, memory, and learning. Glutamate also plays major roles in the development of the CNS, including synapse induction and elimination and cell migration, differentiation, and death (Hansen et al., 2021; Gill and Pulido, 2001; Nedergaard et al., 2002; Hinoi et al., 2004; Niswender and Conn, 2010; Colombo and Francolini, 2019). In addition, glutamate plays a signaling role in peripheral organs and tissues, among them the heart, kidney, intestine, lungs, liver, ovary, testis, bone, pancreas and the adrenal, pituitary and pineal glands, and several other organs and tissues (Gill and Pulido, 2001; Nedergaard et al., 2002; Hinoi et al., 2004).

### 1.2 Glutamate receptors

Glutamate induces its numerous effects via binding and activating a very large number of glutamate receptors (GluRs) (Reiner and Levitz, 2018).

The two main families of GluRs are the ionotropic glutamate receptors (iGluRs) (Hansen et al., 2021) and the metabotropic glutamate receptors (mGluRs) (Niswender and Conn, 2010), each consisting of several types, subtypes, and subunits.

### 1.3 Glutamate and ionotropic glutamate receptors at the neuromuscular junction

Colombo et al. report that glutamate and acetylcholine are the most common neurotransmitters used in the central excitatory synapses and at the neuromuscular junction (NMJ) (Colombo and Francolini, 2019). Glutamate receptors localize postsynaptically at neuromuscular junctions in mice, and glutamatergic transmission occurs at these junctions (Berger et al., 1995; Grozdanovic and Gossrau, 1998; Mays et al., 2009; Personius et al., 2022; Personius et al., 2016). Ettorre et al. (2012) found that glutamatergic neurons induce the expression of functional glutamatergic synapses in primary myotubes and review the past and recent experimental evidences in support of the role of glutamate as a mediating neurotransmitter at the synapse between the motor nerve ending and the skeletal muscle fiber at the vertebrate neuromuscular junction.

Personius et al. studied the role and the neuromuscular glutamate receptors, mainly of the NMDA type, and found that NMDA receptors modulate developmental synapse elimination in mice (Personius et al., 2016). Based on all their findings in that study, the researchers concluded that neuromuscular NMDA receptors play important roles in neuromuscular activity and in elimination of excess synaptic input during development (Personius et al., 2016). Personius et al. found that blockage of neuromuscular iGluRs, either NMDA receptors or AMPA receptors, impairs reinnervation following nerve crush in adult mice (Personius et al., 2022). The researchers also showed the presence of NMDA receptors at the endplate after a nerve crush, documented muscle responses to NMDA after the crush, and found that blocking iGluR of the NMDA or AMPA type, during regeneration, slows polyneuronal innervation and behavioral recovery after a nerve crush. Based on these findings, and additional ones discovered in that study, the researchers concluded that iGluRs play a significant role in promoting recovery after a sciatic nerve crush (Personius et al., 2022).

Despite the multiple studies and findings with regards to glutamate in the NMJ, to the best of our knowledge, no study has shown thus far neither the expression of functional iGluRs on the cell surface of human skeletal muscle cells nor that glutamate by itself can induce direct activating effects on human muscle cells, via their iGluRs.

### 1.4 Glutamate contribution to muscle metabolism

Glutamate is the primary amino acid absorbed by resting and active muscles. It participates in various metabolic pathways in the skeletal muscle, both at rest and during contraction (Personius et al., 2016; Ettorre et al., 2012; Rutten et al., 2005).

Glutamate is known to be delivered to the skeletal muscle via different pathways: it is actively absorbed from the circulation, released by intracellular protein degradation, and synthesized by transamination of the branched-chain amino acids (leucine, isoleucine and valine), which are oxidized in the skeletal muscle (Rutten et al., 2005). Glutamate may also be delivered to the muscles by neurons (Rutten et al., 2005). Glutamate seems to play a determining role in the synthesis of glutathione, the most abundant intracellular antioxidant. During exercise, glutamate plays a central role in energy provision, via different metabolic pathways (Rutten et al., 2005).

### 1.5 Glutamate levels are abnormally reduced in the skeletal muscle in few diseases

Importantly, reduced glutamate levels in the skeletal muscle is a consistent finding in several diseases, and several disease-related factors such as hypoxia and oxidative stress were found to be involved in the disturbed glutamate metabolism (Rutten et al., 2005). In a review entitled “Skeletal muscle glutamate metabolism in health and disease: state of the art,” Rutten et al. presented the knowledge updated to 2005 regarding the metabolic function and regulation of glutamate in skeletal muscle under physiological and pathophysiological circumstances (Rutten et al., 2005).

Engelen et al. found that glutamate levels are reduced in patients with chronic obstructive pulmonary disease (COPD) and reported that altered glutamate metabolism is associated with reduced muscle glutathione levels in patients with emphysema (Rutten et al., 2005; Engelen and Schols, 2003; Engelen et al., 2000).

Recently, Caredio et al. reported that prion diseases disrupt glutamate/glutamine metabolism in the skeletal muscle (Caredio et al., 2024). The researchers found that the GLUL gene is upregulated in the skeletal muscle of prion-infected mice and humans with Creutzfeldt–Jakob disease, leading to disruptions in glutamate and glutamine metabolism and a reduction in muscle glutamate levels (Caredio et al., 2024).

### 1.6 Glutamate receptor GluR3 expression and function

**FIGURE 1:**
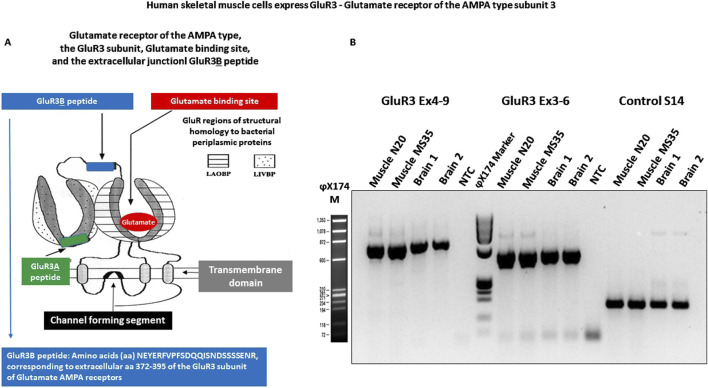
Human primary skeletal muscle cells express GluR3, glutamate receptor of the AMPA-type subunit 3. **(A)** Schematic representation of GluR3. The scheme shows the glutamate receptor of the AMPA-type subunit 3, the natural binding site of glutamate–the physiological neurotransmitter, the approximate location of the extracellular junctional GluR3 **B** peptide (modified from Levite et al. (1999) and from Pin and Bockaert (1995)), and the position of the GluR3A peptide. The scheme is based on Paas et al. (1996). The extracellular “**B**” region of GluR3, also called the “GluR3 **B** peptide,” was previously found to be both a novel agonist binding site (remote from glutamate’s binding site) through which GluR3 can be activated (Cohen-Kashi Malina et al., 2006; Levite et al., 1999; Carlson et al., 1997; McDonald et al., 1999; Twyman et al., 1995) and a self-antigen of autoimmune pathological GluR3 **B** antibodies present in patients intractable epilepsy patients that suffer from “Autoimmune Epilepsy.” The GluR3 **B** peptide is located in between two modular regions of the first GluR3 extracellular domain, suggested to be homologous respectively to the bacterial periplasmic leucine–isoleucine–valine binding protein (LIVBP-like domain) and to the lysine–arginine–ornithine bacterial-binding protein and glutamine-binding protein (LAOBP-/QBP-like domain (Paperna et al., 1996). The GluR3 **B** sequence is one of the two least conserved among the various GluR subtypes (GluR 1–4), as well as across GluRs from different species (Paperna et al., 1996). **(B)** Human skeletal muscle cells express GluR3 RNA. The figure shows that human primary skeletal muscle cells (myoblasts) express GluR3 RNA. The PCR was performed on cDNAs of two unrelated muscle cell samples (N20 and MS35) to span 2 overlapping regions, from exon 3 to exon 6, and from exon 4 to exon 9. Normal brain cDNA was used as a positive control for GluR3. Ribosomal cDNA amplified with specific S14 primers was used as an internal control. NTC is no template DNA. The DNA base-pair ladder used is PhiX (NEB, USA).

The current study focuses only on GluR3, the ionotropic glutamate receptor of the AMPA GluR3 type subunit 3, encoded by the GRIA3 gene. Figure 1A shows a schematic drawing of AMPA GluR3. AMPA receptors that contain the GluR3 subunit can be either homomeric GluR3(o) receptors, heteromeric GluR3(o)/GluR2(o), or GluR3(o)/GluR2(i) receptors (Hansen et al., 2021).

GluR3 is best known for its expression and function in cells of the central nervous system, mainly in neurons (excitatory neurons and interneurons, and to a lesser degree in glia/astrocytes in the CNS. In addition, a number of studies have reported GluR3 expression in other cell types, primarily in T cells of various types and origins (Ganor et al., 2003; Ganor et al., 2009; Ganor and Levite, 2014; Ganor et al., 2007; Levite et al., 2020) (see more on this topic in a separate subsection below) and podocytes (kidney glomerulus (Li et al., 2019).

In the nervous system, GluR3 is involved in the regulation of neural networks. GluR3 is expressed in the spinal cord, brainstem, thalamus and cortex (Engelen et al., 2000; Caredio et al., 2024; Ganor et al., 2003; Ganor et al., 2009). According to Steenland et al. (2008), these GluR3 expression sites suggest that GluR3 regulates functions spanning the neuraxis. Furthermore, Steeland et al. predicted that the localization of GluR3 subunits at respiratory control regions (Sato et al., 1993) and cardiac control regions (Corbett et al., 2003) implies that GluR3 may have an impact on cardiorespiratory function (Steenland et al., 2008). It is also predicted that GluR3 subunit knockout will affect motor tone as these receptors/subunits are located on motor neurons of the ventral spinal cord (Sato et al., 1993). Strikingly, Steenland et al. found that GluR3^−/−^ knockout mice virtually lack electroencephalographic and signatures of NREM sleep and that three of nine GluR3 (−/−) mice expressed seizure activity during wakefulness and sleep (Steenland et al., 2008).

These findings suggest that deletion of the GluR3 gene by itself may predispose to seizures. Moreover, GluR3 gene knockout also produced state-dependent respiratory modulation, with a selective reduction in the breathing rate during behavioral inactivity (Steenland et al., 2008).

Within the brain, Moga et al. found GluR3 immunoreactivity in all pyramidal neurons and astrocytes and in most interneurons (Moga et al., 2003). Selective GluR3 expression is significantly elevated in the somata of parvalbumin-containing interneurons, which are potent inhibitors of cortical pyramidal neurons, and are vulnerable in brains of epilepsy patients (Moga et al., 2003). Taken together, these findings indicate that GluR3 has diverse neurophysiological impacts and is involved in sleep, wakefulness regulation, breathing, and generation of cortical seizures (Steenland et al., 2008).

### 1.7 GluR3 expression in T cells and the direct activating effects of glutamate on T cells

We previously found that normal human as well as human T cell lymphoma and autoimmune T cells express functional GluR3 (Ganor et al., 2003; Ganor et al., 2009; Ganor and Levite, 2014; Ganor et al., 2007; Levite et al., 2020).

We also discovered that glutamate activates directly its GluRs expressed in T cells and induces/increases all these T cell functions and features: integrin-mediated adhesion to laminin and fibronectin, chemotactic migration, elevation of CD147/EMMPRIN (a cancer-associated matrix metalloproteinase (MMP) inducer, MMP-9 secretion, engraftment of cells *in vivo*, CD3 zeta expression, and most importantly T cell killing of cancer cells (Ganor et al., 2003; Ganor et al., 2009; Ganor and Levite, 2014; Levite et al., 2021; Saussez et al., 2014). We also found that glutamate reduces PD-1 expression in T cells (Levite et al., 2021). All these findings indicate that glutamate and GluR3 seem to have very important roles in T cell functions and in the entire function of the immune system.

### 1.8 GluR3 as an autoantigen for pathogenic autoimmune GluR3 antibodies in “Autoimmune Epilepsy”

Apart from being an important physiological glutamate receptor/subunit, GluR3 is an antigen of very detrimental autoimmune GluR3 antibodies (Levite and Goldberg, 2021). Autoimmune GluR3 antibodies play a key pathological role in “Autoimmune Epilepsy” (Levite et al., 2020; Levite and Goldberg, 2021; Levite, 2002). In our own multiple studies on “Autoimmune Epilepsy,” and in studies of other groups, autoimmune GluR3 antibodies, especially GluR3 **B** antibodies directed against the extracellular junctional and antigenic peptide “**B”** peptide of GluR3 (shown schematically in Figure 1A), were found to be pathological antibodies that cause multiple detrimental effects *in vitro* and *in vivo*. In particular, the GluR3 **B** antibodies can kill neural cells by three mechanisms: induction of excitotoxicity, reactive-oxygen-species (ROS), and/or complement-fixation. The GluR3 **B** antibodies were also found to induce or facilitate in animal models all the following effects: brain damage, seizures, and behavioral impairments (for the most recent review, see Levite and Goldberg (2021)), and for some of the original research papers, see Levite et al. (2020), Cohen-Kashi Malina et al. (2006), Levite et al. (1999), Levite and Hermelin (1999), Ganor et al. (2004), Ganor et al. (2014), Ganor et al. (2005a), Ganor et al. (2005b), Goldberg-Stern et al. (2014).

The autoimmune GluR3**B** peptide antibodies were found so far in the serum of ∼27% of >300 persons with severe, intractable, and enigmatic epilepsy of various types (for the most updated review and original paper that summarize and cite all the relevant studies see Levite and Goldberg (2021); Taiwo et al. (2025). In addition, in a recent study, we found that epileptic Nodding Syndrome (NS) patients have autoimmune GluR3 **B** antibodies and that the NS patient’s GluR3 **B** peptide autoimmune antibodies bind both neural cells and immune T cells (that express GluR3), induce ROS in both neural cells and T cells, and kill both of these cell types (Levite et al., 2020).

NS is a form of epilepsy affecting children in Sub-Saharan Africa, marked by head-nodding seizures, progressive cognitive decline, growth stunting, and developmental delays (Abd-Elfarag et al., 2021; Samia et al., 2022). NS is now recognized as an idiopathic epileptic encephalopathy with tauopathy. NS is strongly associated with muscle wasting (loss of muscle mass) and low muscle mass and can involve a temporary loss of neck muscle tone (Abd-Elfarag et al., 2021; Samia et al., 2022).

The characteristic head nodding results from a temporary loss of neck extensor tone. While muscle strength is generally maintained, the disorder can progress to include peripheral muscle wasting and generalized wasting. The head nodding, which can occur multiple times per minute, is thought to be caused by a loss of muscle tone in the neck and potentially other muscle groups (Abd-Elfarag et al., 2021; Samia et al., 2022).

Recent studies show that autoimmunity, the parasite *Onchocerca volvulus*, possible environmental triggers, and vitamin B6 deficiency contribute to NS. While treatments for symptoms exist, an effective cure is lacking. Current clinical trials test the effectiveness of doxycycline as a treatment for NS in children and adolescents (Idro et al., 2024).

In the present study, we asked three main questions: do human skeletal muscle cells express functional GluR3, are these cells activated by glutamate, and do autoimmune GluR3 **B** antibodies of either NS patients or other intractable epilepsy patients bind and affect human skeletal muscle cells.

The novel findings of this study provide a positive answer to all of these questions. However, further studies in various directions are of course needed to confirm, expand, and deepen these findings and to understand and examine their implications for muscle cells and motor function of healthy or diseased individuals.

## 2 Materials and methods

### 2.1 Human primary skeletal muscle cells (myoblasts)

The primary culture of human muscle cells (myoblasts) was derived from the gastrocnemius of a 32-year-old female following an isolation procedure as described (Amsili et al., 2007). The culture was obtained from the Muscle Tissue Culture Collection (MD-NET, service structure S1, 01GM0302, BMBF, Eurobiobank) at the Friedrich-Baur Institute. The biopsy material was obtained from the Ludwig-Maximilians Universitat Munchen (Germany. IRB approval no 45-14-2014). The primary muscle cells were cultured in Promocell Human muscle growth medium (PromoCell, C-23160). The medium was changed every 3 days. The cells were used in experiments as myoblasts. In IncuCyte experiments, however, the medium was not changed during the entire incubation time, as indicated for each experiment, and from days 3 to 4 on culture, their morphology pointed to initiation of differentiation in spite of the fact that a differentiation medium was not used.

### 2.2 The South-Sudanese Nodding Syndrome patients and healthy subjects whose antibodies we studied and the related IRB approval

The Ministry of Health of South-Sudan had provided us an allowance to conduct a clinical diagnostic study, and withdrawal of small blood samples for diagnostic research, from South-Sudanese Nodding Syndrome patients and healthy subjects, based on an IRB approval provided 25 May 2012. This investigation (both cohorts) was performed at the town of Mundri, Western Equatoria, South-Sudan. All South Sudanese NS patients and control healthy signed an informed consent.

The relevant clinical information on the NS patients included in the present study is depicted in our previous study on the presence and pathogenic effects of GluR3B antibodies in these NS patients (Levite et al., 2020).

All the NS patients in the study group were diagnosed by a pediatric neurologist. For some of them, a deeper diagnosis was performed, and most of these clonic–tonic seizures and additional clinical symptoms were documented. Healthy South Sudanese subjects, at similar age range, ratio between males and females, and geographical locations to that of the NS patients, were recruited for the study. A small volume of blood was withdrawn from all the NS patients and healthy subjects by the Sudanese clinicians, serum was separated in place, and all samples were shipped to Israel for all the subsequent *in vitro* and *in vivo* studies described in this paper and in Levite et al. (2020).

### 2.3 The intractable epilepsy patients whose antibodies we studied, and the related Helsinki approval

The present study on the antibodies of intractable epilepsy patients received an IRB approval No. 0339-09 from the ethic committee of Rabin Medical Center, Israel, which the Schneider Medical Center in Israel is affiliated to. The epilepsy patients signed informed consent forms. All the studied epilepsy patients are/were treated by Prof. Hadassa Stern Goldberg (author), Head of the Epilepsy Unit in Schneider Medical Center. Table 1, showing the epilepsy patient’s clinical information, was prepared by Prof. Hadassa Stern Goldberg. The epilepsy patients are addressed throughout the study in coded names, and the manuscript does not disclose any confidential information about the patients, which can help identify them.

**TABLE 1:** Clinical information on the Intractable epilepsy patients whose antibodies were studied in the present study.

Epilepsy patient code	Male/Female	Year of birth	Current age	Age of seizure onset (months)	Type of epilepsy	Etiology of epilepsy	Epilepsy duration (years)	Current AED	Mental retardation	Psychiatric/behavioral problems	Motor/Gait problems	Brain MRI	EMG
IE-2	Male	2009	24	6	Focal+generalized	Dravet syndrome (SCN1A mutation*)	23.5	VPA, Fenfluramine, VNS, Cannabidiol	YES	YES	No**	Normal	Not done
IE-3	Female	1998	25	72	Generalized	Unknown	19	VPA, LEV, VNS, Cannabidiol	YES	YES	Bedridden	Abnormal (atrophy)	Not done
IE-4	Female	2009	16	48	Focal	Rt. FrontTemporal	12	Lamictal, TPM, Lacosamide	YES	YES	No**	Increased T2 signals in subcortical gray white junction	Notdone
IE-6	Female	1993	30	6	Generalized	Dravet syndrome (SCN1A deletion*)	29.5	VPA, LEV, CLZ, VNS	YES	YES	No**	Normal	Notdone
IE-7	Male	1998	25	11	Focal	Probably genetic (Unknown mutation)	24	Trileptin, VPA, CLZ, VNS	YES	YES	Yes, Wheelchair	Normal	Axonal and demyelinative motor and sensory polyneuropathy
IE-13	Male	1993	29	12	Generalized	Dravet syndrome (SCN1A mutation)	28	Sulthiam, CLZ, TPM, VPA	YES	YES	No**	Normal	Not done
IE-14	Male	1996	27	12	Generalized	Dravet syndrome (SCN1A mutation*)	12	VPA, LEV, CLZ, Cannabidiol	YES	YES	Unknown**	Normal	Not done

AED - Antiepileptic drugs, VPA - Valproic acid, LEV - Levetiracetam, CLZ - Clobazam, TPM – Topiramate, VNS - Vagal nerve stimulation, EMG – Electromyogram, MRI - Magnetic resonance imaging.

*The SCN1A gene - Sodium voltage-gated channel alpha subunit 1. The SCN1A gene belongs to a family of genes that provide instructions for making sodium channels that transport positively charged sodium ions into cells, play a key role in a cell's ability to generate and transmit electrical signals. Hundreds of mutations in the SCN1A gene have been found to lead to loss-of-function, and known to cause a spectrum of epilepsy disorders and other neurological conditions, primarily Dravet Syndrome (also known as severe myoclonic epilepsy in infancy), and genetic epilepsy with febrile seizures plus (GEFS+).

**A ‘No’ answer indicates the absence of motor problems in a given epilepsy patient, but it is only true for the patient's age at the time of the study. In Dravet syndrome, gait problems begin at a later age.

### 2.4 Reverse transcription end-point PCR for GluR3

RNA was extracted from muscle cells by the Tri Reagent isolation solution (Cat# TR 118, Molecular Research Center, United States). Brain1 RNA was purchased (Thermofisher Cat #AM7962). Brain2 RNA was a kind gift from Dr Eisenberg. cDNA synthesis was performed by reverse transcription of the RNAs using the M-MLV Reverse Transcriptase kit (Cat#M1701, Promega, WI) according to the manufacturer’s protocol and screened for GluR3 by PCR and sequencing. Two sets of primers were used, at a concentration of 0.4 mM/reaction, as described in Ganor et al. (2003); the first, spanning exons 3 to 6: upstream primer (GluR3 E3), GACGCAGATGTGCAGTTTGTCATC; downstream primer (GluR3 E6), TAGTGGTGCATTCTTGGCTTCAGG, resulting in a 516-bp product; the second, spanning exons 4 to 9: upstream primer (GluR3 E4), CGATACTTGATTGACTGCGA; downstream primer (GluR3 E9), TACTATGGTCCGATTCTCTG, resulting in a 632-bp product As an internal control for the integrity of the samples, a ribosomal RNA S14 region was amplified using the following primers: upstream primer, GTCCATGTCACTGATCTTTCTGGC; downstream primer, GTTTGATGTGTAGGGCGGTGATAC, resulting in a 166-bp product.

Conditions for PCR were as follows: 94 °C for 1 min, 60 °C for 40 s, and 72 °C for 40 s (29 cycles for S14 PCR and 38 cycles for GluR3 PCR). The cDNA sequencing was performed with an automated sequencer at the sequencing unit of the Hebrew University of Jerusalem.

### 2.5 Mouse GluR3B monoclonal antibody

The mouse anti-human/rat/pig GluR3 **B** peptide monoclonal antibody (mAb) was originally produced owing to the request of the research group of M.L. (first author herein), at the Life Science Core Facilities, Faculty of Biochemistry, Weizmann Institute of Science, Israel. Since then, the GluR3**B** mAb has been used successfully for several published studies, among them Levite et al. (2020), and commercialized by the Weizmann Institute of Science to Medimab, Canada (Medimab, Cat no. #GLU149.29.61).

### 2.6 The GluR3B peptide used for detection and affinity-purifications of the GluR3B antibodies

The GluR3 **B** peptide (also called the ‘**B**’ region of GluR3) is a 24-amino acid peptide, whose amino acid (aa) sequence is NEYERFVPFSDQQISNDSSSSENR), corresponding to aa372-395 of the glutamate/AMPA receptor/subunit GluR3. The GluR3 **B** peptide used in ELISA in Levite et al. (2020) was synthesized by Thermo Fisher Scientific. The biotinylated GluR3 **B** peptide Biotin - GSGSNEYERFVPFSDQQISNDSSSSENR-OH, used herein for affinity-purification of human GluR3 **B** antibodies from the era of NS patients was synthesized by Pepscan, Netherlands.

### 2.7 Detection by ELISA of autoimmune GluR3B antibodies

Sera of the epilepsy patients and healthy individuals were tested for the presence of GluR3**B** antibodies by ELISA, as done previously in several of our studies (see, for example, Goldberg-Stern et al. (2014)). In the first (coating) step, microtiter wells of a Maxisorp microtiter immunoplate (Nunc, Roskilde, Denmark) were covered with 50 µL per well of 10^-7^ M of GluR3B peptide suspended in a coating buffer of 0.1 M NaHCO3 pH 8.2 for detecting the respective GluR3**B** antibodies. In parallel, other microtiter wells of another control microtiter plate were covered with 50 μL per well of phosphate-buffered saline (PBS) with 1% bovine serum albumin (BSA) only (i.e., without any antigenic peptide), to detect nonspecific control binding to BSA.

The two microtiter plates were incubated overnight at 4 °C. The following day, the microtiter wells of each plate were washed twice with 100 µL of PBS with 1% BSA per well and then once with 100 µL per well of double-distilled water (DDW). The next step was the blocking of the nonspecific binding, performed by adding 50 µL per well of PBS+1%BSA to all the microtiter wells, and subsequent 2-h incubation at room temperature (RT). At the end of the blocking stage, the microtiter wells were washed, first with PBS with 1% BSA and then with DDW, as in the previous washing step. Thereafter, the serum of each patient or control individual underwent serial dilutions in PBS with 1% BSA, to final dilutions of 1:10, 1:100, and 1:1,000. Then, 100 µL of each serum at each of the three dilutions was added to two adjacent duplicate microtiter wells, in each of the two plates: the GluR3 **B**-coated and the PBS+1%BSA-coated plates. The two microtiter plates were then incubated overnight at 4 °C. The next day, the wells were washed once more, first with PBS+1%BSA and then with DDW, as in the previous washing steps. Thereafter, horseradish peroxidase (HRP)-conjugated goat anti-human IgG (Jackson Immunoresearch, West Grove, PA) was diluted in PBS+1%BSA to a final dilution of 1:1,000, and 50 µL of this dilution was added to each well in the microtiter plates. Then, the plates were incubated for 2 h at RT. Following an additional washing step, as described above, ABTS peroxidase substrate (Kirkegaard and Perry Laboratories, Gaithersburg, MD) was added to each well, and the optical density (OD) at 405 nm was measured with an ELISA reader. Repeated OD measurements of the plates were performed 3–4 times, at approximately 20-min intervals, from the time the substrate was added. The first OD reading was done when the green color in some wells became clear.

To obtain the level of the specific GluR3 **B** peptide antibodies in each serum, a two-step calculation was performed as follows: 1. Calculation of the average value of every set of adjacent duplicate wells, 2. Subtraction of the average OD of the nonspecific binding to PBS+1%BSA of each serum sample, in each dilution and each OD reading, from the average OD of the specific binding of that specific serum to the GluR3 **B** peptide, of the very same serum sample, at the same dilution, and the same OD reading.

Thus, the final values of specific GluR3 **B** peptide antibodies were determined for each serum dilution using the equation: [average OD of duplicate wells in the GluR3 **B**-coated plate] - [average OD of duplicate wells in the PBS+1%BSA -coated plate]. In most cases, the results of the second OD reading (∼30 min after adding the substrate) were the clearest.

Findings were considered positive if the final value of specific GluR3 **B** peptide antibodies was equal or higher than the experiment’ cut off. The cutoff was the calculated value of the average OD + 2 X SD of the GluR3 **B** antibodies detected in all the healthy control individuals, tested in the same experiment. In most experiments, the calculated cutoff was approximately 0.25–0.4 OD.

### 2.8 Affinity purification of human GluR3B antibodies from serum of nodding syndrome patients and of healthy control eluents of healthy subjects

The protocol consisted of the following steps: 1. Equilibration of 150-µL packed beads streptavidin–agarose (Thermo Scientific Pierce, Cat no. 20353) with PBS on 2-mL open columns. 2. Loading on columns and reloading four times 1 mg of biotinylated peptide resuspended in 2 mL PBS. 3. Washing peptide affinity column with 5 × 1 mL PBS before peptide specific antibody purification. 4. Diluting 0.5 mL serum in 2 mL PBS, spinning 10 min, 13,000 rpm and discharging pellets. 5. Loading on the peptide affinity column and reloading unbound another four times. 6. Washing with 1 mL PBS several times up to reaching OD 280 nm of washing <0.05 OD280 (blank PBS). 7. Adding 250-µL elution buffer (0.1 M acetic acid pH 3.0) and then fitting a stopper to the column, followed by waiting for approximately 2 min at 4 °C and spinning. 8. Collecting the 250 µL on a tube containing 100 µL 1M TrisHCl pH 8.5 (in order to neutralize the solution immediately). Repeating five times. 9. Checking the protein concentration by OD 280 nm, and pooling IgG-rich fractions. 10. Dialyzing vs. 100 mL PBS, changing buffer after 2 h, and final overnight dialysis vs. fresh PBS. 11. Aliquoting and freezing purified antibodies.

### 2.9 Purification of IgG from the serum of nodding syndrome patients, intractable epilepsy patients, and healthy subjects of two respective control groups

Sera of South Sudanese NS patients and of intractable epilepsy patients (see Table 1) and respective healthy control subjects were centrifuged at 1,600 RPM for 10 min 4 °C. Clean supernatants were diluted to final 4 mL with PBS (10 mM NaPO_4_, 150 mM NaCl pH7.4). The samples were loaded onto a PBS equilibrated 1 mL Protein G Sepharose FF (GE Healthcare) affinity chromatography column using AKTA AVANT (GE Healthcare). Then, the column was washed with PBS until reaching low OD 280 nm; IgG was eluted with 0.1 M glycine pH 3.0 buffer and 1 mL eluted fractions were collected in tubes containing 0.08 mL 1.5 M TriHCl pH 8.5 in order to get immediate pH neutralization. IgG fractions were pooled according to the OD 280 nm profile and dialyzed 3 h vs. 100 mL PBS and ON 4 °C using fresh PBS using 13-kDa cutoff dialyze tubes. Finally, they were concentrated by ultrafiltration devices (cut-off 30 kDa of Amicon), and purified IgG samples were analyzed for contents by A280 (E.C 1.35) and by SDS-PAGE (Novex 4%–12%, Thermo), using reduced and non-reduced sample buffer. PageRuler (Thermo) used as MW markers. Purified IgG was aliquoted and frozen.

### 2.10 Immunofluorescence staining and confocal microscopy of human skeletal muscle cells for detecting the binding of mouse GluR3B mAb to human skeletal muscle cells

Immunostaining of GluR3 was done in two steps. First, fixed human skeletal muscle cells (growing on slides) were incubated with a mouse GluR3 **B** mAb (which we previously produced specifically against the isolated GluR3 **B** peptide, corresponding to GluR3 aa 372 to 395, and showed later that it binds the GluR3 **B** peptide in its natural configuration in human neural cells and T cells (Ganor et al., 2007; Levite et al., 2020).

Second, the human skeletal muscle cells were immunostained with anti-desmin antibody to confirm they were indeed muscle cells and with DAPI to detect intact cells.

### 2.11 Immunostaining and confocal microscopy for detecting binding of nodding syndrome patients’ affinity-purified GluR3B antibodies or IgGs of other intractable epilepsy patients that have elevated GluR3B antibodies to human skeletal muscle cells

Human primary skeletal muscle cells (growing on slides) were treated along the following steps (and three washes were performed in between key steps): 1. Fixation with 4% paraformaldehyde; 2. Incubation with affinity-purified GluR3 **B** antibodies of NS patients or healthy subjects or with purified IgGs of intractable epilepsy patients (Table 1) or respective control healthy subjects (either 1 h in RT, or overnight (ON) in 4C); 3. Blocking with PBS 2% BSA (30 min RT); 4. Incubation with a secondary Cy3- Goat and human F (ab)_2_ fragment (30 min RT).

### 2.12 Immunofluorescence staining and confocal microscopy for detecting *in vitro* killing of live human skeletal muscle cells by IgG antibodies of intractable epilepsy patients

Primary human skeletal muscle cells *(myoblasts)*, growing on slides, were treated along the following steps (and three washes were performed in between key steps): 1. Incubation with total human IgG (∼0.2 mg/mL) of either intractable epilepsy patients or a healthy control subject (either 1 h in RT or ON in 4C); 2. Blocking with PBS 2% BSA (30 min RT); 3. For staining dead cells: Incubation with Sytox green (ThermoFisher Scientific’ 1 μM, 30 min, RT); 4. Fixation with 4% paraformaldehyde; 5. Blocking with PBS 2% BSA (30 min RT); 6. Incubation with a secondary Cy3- Goat and human F (ab)2 fragment (Jackson ImmunoResearch code: 109-166-088) (30 min RT); 7. Permeabilization and further blocking (0.2% Triton, 2% BSA in PBS, 30 min, RT); 8. Finally, to confirm they were indeed muscle cells, the human skeletal muscle cells were immunostained with mouse anti-human desmin mAb and then with goat anti-mouse IgG-Alexa 647. At the last stage, the cells were stained with DAPI to detect intact cells.

### 2.13 Usage of the IncuCyte device and technology for continuous and real time follow-up, photography, and analysis of human primary skeletal muscle cells growing in tissue culture

#### 2.13.1 The IncuCyte system

The IncuCyte® Live-Cell Analysis System (Sartorius) is a continuous live cell imager.

It is a flexible assay platform that sits inside a standard tissue culture incubator and automatically acquires and analyzes phase and fluorescent images for extended period of time. The IncuCyte is designed to efficiently capture cellular changes in the incubator, and all the possibilities and modes of using it for various biological purposes can be found in https://www.sartorius.com/en/products/live-cell-imaging-analysis.

#### 2.13.2 Continuous follow-up of human primary skeletal muscle cells in tissue culture

Primary human skeletal muscle cells (myoblasts), growing in tissue culture in Promocell Human muscle growth medium (PromoCell, C-23160), were seeded evenly in wells of microtiter plates (either 48 or 24 well plates) and then inserted into the IncuCyte incubator (IncuCyte® S3 Live-Cell Analysis System, Cat# No. 4763, Sartorius, Israel). Then, the IncuCyte software was set up to measure continuously the cells’ confluence (and their fluorescence in case they were fluorescently labeled before the onset of the experiment) and to photograph the cells in nine fields of every single microtiter well, every 3 h, for several days, as pointed out for each experiment. The analysis of the quantitative data was done automatically by the IncuCyte analysis software. The quantitative kinetic curves were also drawn by the IncuCyte software based on the analyzed data. The later calculations of the averages, SD and p values and the drawing of the histograms at chosen time points were done in Excel, after exporting the raw data from the IncuCyte to Excel.

### 2.14 Using the IncuCyte follow-up system and the fluorescent dye CoroNa™ green to study the changes in the levels of intracellular sodium (Na^+^) ions in human primary skeletal cells

Human primary skeletal cells (myoblasts), growing in tissue culture plates, were loaded with CoroNa™ Green, AM, cell permeant dye (Molecular Probes and ThermoFisher Scientific Cat. No 36676) according to the manufacturer instructions. The CoroNa Green dye is a Sodium ion (Na+) indicator that exhibits an increase in green fluorescence emission intensity upon binding sodium (Na+ ions). After loading the muscle cells with CoroNa™ Green, the cells were distributed evenly in wells of microtiter plates, and the plates were placed inside the IncuCyte incubator, for few hours of rest, before starting the experiment/s.

Then, we set up the IncuCyte system to photograph the skeletal muscle cells, (both phase contrast and corona green fluorescence) in all the microtiter wells in the plate (according to the early planning) few times. Few hours later, we started the experiment: we quickly and gently removed the microtiter plate from the IncuCyte incubator, placed it in an adjacent sterile hood, and added either glutamate, AMPA, NMDA, or IgG of a given epilepsy patient or healthy subject, at the desired concentration (all prepared freshly from frozen stocks of much higher concentrations just before each experiment) to replicate microtiter wells in the right predetermined position in the plates. Immediately afterward, we returned the microtiter plate very gently to the IncuCyte and photographed the very same wells again (both Phase contrast and CoroNa™ Green Fluorescence). Thereafter, in the long-term experiments, the IncuCyte was designed to measure, analyze, quantify both the confluence and the Corona Green Fluorescence, proportional to the levels of intracellular Sodium ions, and to photograph the cells every 3 h. In the short-term (responses to glutamate within 2 min), experiment whose representative photos are shown in Figure 9, the time that elapsed between the first photography (before addition of any molecule), and the second photograph was exactly 2 min after addition of either glutamate, AMPA, NMDA or human IgGs of a given epilepsy patient or of healthy control subject.

### 2.15 Statistical analysis

Statistical analysis was performed using the Student’s t-test. The p values are written inside the figures and/or specified in the “Results” in all experiments in which the statistical analysis could be performed.

## 3 Results

### 3.1 Human primary skeletal muscle cells express authentic GluR3 RNA

RNAs of primary human skeletal myoblasts growing in tissue culture were tested for their expression of GluR3, glutamate receptor of the AMPA-type subunit 3, by GluR3-specific RT-PCR, using two sets of GluR3-specific primers, spanning exons 3–9 of the gene (Ganor et al., 2003). As a positive control, human brain RNA was tested in parallel since GluR3 is highly expressed in several areas of the brain (Ganor and Levite, 2014; Levite et al., 2020). As seen in Figure 1B, we found that the human primary skeletal muscle cells express GluR3 RNA.

### 3.2 Human skeletal muscle cells express the GluR3 protein and its extracellular junctional GluR3B peptide on their cell surface

**FIGURE 2:**
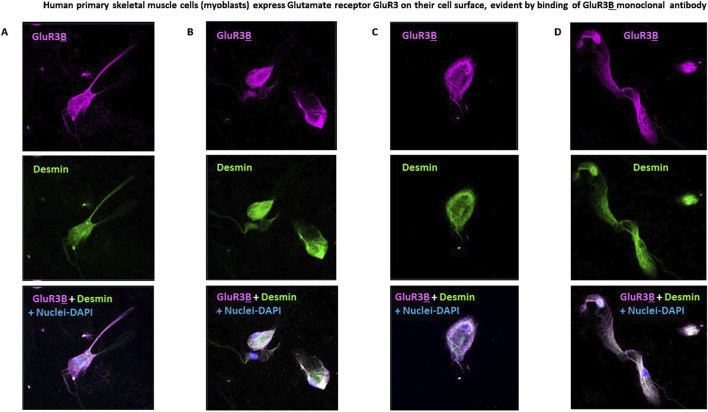
Human primary skeletal muscle cells express glutamate receptor GluR3 on their cell surface, evident by binding of the GluR3B monoclonal antibody. **(A–D)** Representative confocal microscopy photos of four fields of human primary skeletal muscle cells (myoblasts) growing in tissue culture and immunostained with 1. Mouse anti-GluR3 **B** mAb (seen in pink in the upper panels and in the lower panels showing overlaps of all the staining); 2. Anti-human desmin mAb (shown in green in the middle panels and in the lower overlap panels). Desmin is a muscle-specific protein and therefore confirms the muscle origin of the studied primary skeletal muscle cells; 3. DAPI that stains the cell’s nucleus and allows detection and photography of valid cells (seen in blue in the lower overlap panels).

Next, we found that primary human skeletal muscle cells (myoblasts) express GluR3 and its extracellular junctional GluR3 **B** peptide (shown schematically in Figure 1A) on their cell surface. This is demonstrated by the specific binding and immunostaining of the skeletal muscle cells by a mouse GluR3 **B** monoclonal antibody (mAb) and subsequent confocal microscopy images (Figures 2A–D).

### 3.3 GluR3B monoclonal antibody increases the number of human skeletal muscle cells growing in tissue culture

To find out if GluR3 is an active functional receptor in human skeletal muscle cells, we had to target it selectively, without affecting other GluRs. However, to the best of our knowledge, there is no selective GluR3 receptor agonist or antagonist, and therefore none could serve for targeting only GluR3. There are only glutamate receptor agonists and antagonists that bind to a broad family of GluRs, including among them AMPA and NMDA agonists and antagonists.

In the absence of the selective GluR3 receptor agonist, we decided to use the mouse GluR3 **B** mAb and test if it affects the skeletal muscle cells. Our hypothesis was that the GluR3 **B** mAb may activate GluR3 since it was previously found that the GluR3 **B** peptide defines a novel GluR subunit-specific agonist binding and activation site within GluR3, remote and independent of the “classical” glutamate binding site, and that GluR3 **B** antibodies can activate GluR3 by binding this “**B”** region (Cohen-Kashi Malina et al., 2006; Levite et al., 1999; Carlson et al., 1997; McDonald et al., 1999; Twyman et al., 1995).

**FIGURE 3:**
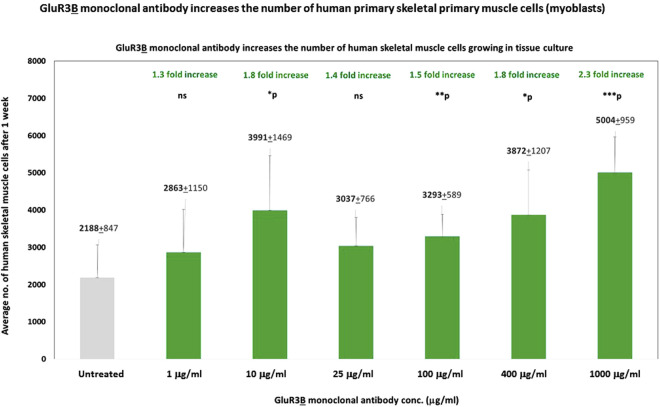
GluR3B monoclonal antibody increases the number of human primary skeletal muscle cells growing in tissue culture. Mouse GluR3 **B** mAb at six different concentrations: 1, 10, 25, 100, 400, or 1,000 μg/mL in PBS, or control PBS only, was added to human primary skeletal muscle cells (myoblasts) growing in the tissue culture. The cells were monitored and photographed using the IncuCyte system (as described in the Results and Materials and Methods). After 1 week, the cells were counted by flow cytometry. Each experimental group consisted of six replicate wells (n = 6). The figure shows the average numbers and the standard deviation (SD) of the muscle cell number in each experimental group and the statistical values calculated by T. test. The p values show that the effect of the GluR3 **B** mAb were statistically significant when added at a conc. of either 10, 25, 100, 400, or 1,000 μg/mL. The statistical analysis comparing GluR3 **B** mAb-treated muscle cells to untreated cells yielded the following p values: GluR3 **B** mAb 1 μg/mL - p = 0.13 - ns; 10 μg/mL - *p = 0.02; 25 μg/mL - *p = 0.06 - ns; 100 μg/mL - **p = 0.006; 400 μg/mL - *p = 0.02; 1,000 μg/mL (=1 mg/mL) - ***p = 0.001. ns = not significant.

We found that a single addition of GluR3 **B** mAb increased, in a dose-dependent manner, the number of human skeletal muscle cells growing in the tissue culture for 1 week (Figure 3). The GluR3 **B** mAb was added to the skeletal muscle cells only once at different concentrations: 1 μg/mL, 10 μg/mL, 25 μg/mL, 100 μg/mL, 400 μg/mL, or 1 mg/mL and found to increase the cell number by: 1.3-, 1.8-, 1.4-, 1.5-, 1.8-, and 2.3-fold, respectively (Figure 3). We speculate (but did not prove directly) that the GluR3 **B** mAb induced this effect by activating and inducing *de novo* proliferation of the skeletal muscle cells.

**FIGURE 4:**
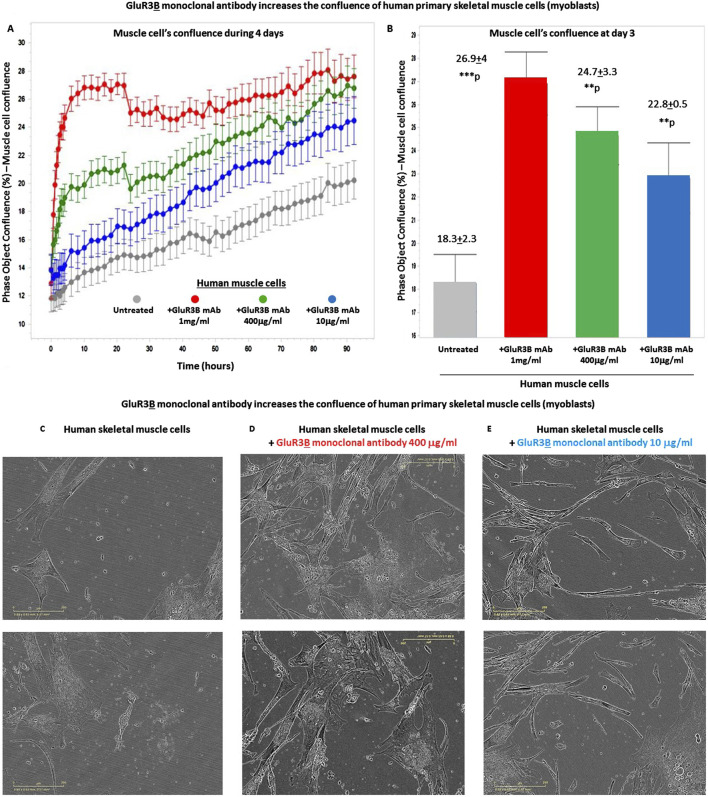
GluR3B monoclonal antibody increases the confluency of human primary skeletal muscle cells growing in tissue culture. **(A)** The graphs of the confluency kinetic curves of the human primary skeletal muscle cells (myoblasts) growing in tissue culture inside an IncuCyte incubator and connected to the IncuCyte system for 4 days. At the onset of the experiment, the muscle cells were either untreated (i.e., added only with PBS for control, gray curve) or added with GluR3 **B** mAb at different concentrations: either 10 μg/mL (blue), 400 μg/mL (green), or 1 mg/mL (red). Each experimental group consisted of six independent replicate wells (n = 6). Thereafter, their confluency was measured and analyzed repeatedly by the IncuCyte system every 3 h. The confluency kinetic curves shown in the figure represent the averages and SD for each group. **(B)** Confluency histogram of the human skeletal muscle cells in the different experimental groups of the same experiment, at day 3. The histograms also show the average, SD, and statistical value of each experimental group. The statistical analysis comparing GluR3 **B** mAb-treated muscle cells to untreated cells yielded the following p values: GluR3 **B** mAb 1 mg/mL - ***p = 0.001; 400 μg/mL - **p = 0.003; 10 μg/mL - **p = 0.003. **(C–E)** Representative photos (taken by the IncuCyte) of the human primary skeletal muscle in each experimental group, at day 4 of the same experiment. The photos of the untreated muscle cells are shown in **(C)**, those of the cells added with the GluR3**B** mAb at 400 μg/mL are seen in **(D)**, and those of the cells added with the GluR3**B** mAb at 10 μg/mL are seen in **(E)**.

Next, we tested if the GluR3 **B** mAbs increase the confluence of the human skeletal cells growing in the tissue culture and used the IncuCyte technology for studying that. The positive results are shown in Figure 4. As a reminder: in general, in cell culture biology, confluence refers to the percentage of the surface of a culture dish that is covered by adherent cells, while the cell number refers to, trivially, the number of cells in a given region. Cell confluency can affect cell behavior and growth, so it is important to accurately and reliably measure cell confluency. For further facts on the importance of cell confluence, please refer to https://bitesizebio.com/63887/cell-confluency/.

We found that a single addition of GluR3 **B** mAb, at either 1 mg/mL, 400 μg/mL or 10 μg/mL, increased significantly the confluence of the skeletal muscle cells during few days. This finding is observed in the following figures: Figure 4A, that presents muscle cells’ confluence curves of all the experimental groups during 4 days from addition of the GluR3**B** mAb; Figure 4B, that presents histograms of the confluence level at day 3 and also the averages, SD, and statistical values of the experimental groups at this day; Figures 4C–E, that show representative photographs of the skeletal muscle cells in this experiment, taken by the IncuCyte microscope and camera at day 4. The representative photographs of the untreated muscle cells are seen in Figure 4C, the photographs of the muscle cells added with 400 μg/mL GluR3 **B** mAb are seen in Figure 4D, and the photographs of cells added with 10 μg/mL GluR3 **B** mAb are seen in Figure 4E. These images visually illustrate the confluence-increasing effect of the GluR3 **B** antibody.

### 3.4 Glutamate increases the number of human skeletal muscle cells growing in tissue culture

**FIGURE 5:**
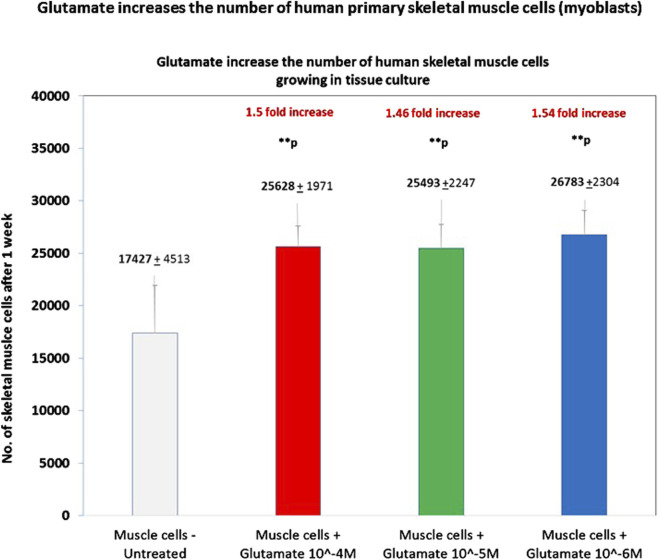
Glutamate increases the number of human primary skeletal muscle cells. The figure shows the number of human primary skeletal muscle cells (myoblasts) after 1 week from the onset of an experiment in which we tested if glutamate by itself can induce proliferation of these cells. At the onset of the experiment, the muscle cells were either untreated (i.e., added only with PBS for control) or added with glutamate at three different concentrations: 10^−4^M, 10^−5^M, or 10^−6^M in PBS./After 1 week, the cells were detached from the microtiter wells and counted by flow cytometry. Each experimental group consisted of six replicate wells (n = 6). The figure shows the average ±SD number of cells counted in each experimental group and the statistical values. The figure shows that glutamate at either 10^−4^M, 10^−5^M, or 10^−6^M increased the number of the skeletal muscle cells by ∼1.5 fold and that the effect was significant in all these concentrations. The statistical analysis comparing glutamate-treated muscle cells to untreated cells yielded the following p values: glutamate 10^-4^M - **p = 0.009; glutamate 10^-5^M - **p = 0.001; glutamate 10^-6^M - **p = 0.005.

In the next step, we asked if glutamate, the natural neurotransmitter that binds GluR3 and all other types of GluRs, activates directly primary human skeletal muscle cells (myoblasts). We found that a single addition of glutamate, at either 10^−4^M, 10^−5^M, or 10^−6^M, increased by ∼1.5 fold, in a statistically significant manner, the number of human skeletal muscle cells growing in culture for 1 week (Figure 5). To the best of our knowledge, this is the first evidence that glutamate can independently activate directly human primary skeletal muscle cells and increase their number.

### 3.5 Glutamate increases the levels of intracellular sodium (Na^+^) ions in human primary skeletal muscle cells growing in tissue culture

Next, we performed different functional experiments, testing if glutamate can independently increase intracellular sodium (Na^+^) levels in human myoblasts. We next briefly summarize the background and reason for testing intracellular sodium levels.

Regulation of intracellular sodium (Na^+^) levels and mitochondrial sodium plays an important role in multiple critical cellular processes in health and disease. Sodium concentration in the extracellular fluid is very carefully maintained in a range of 135–145 mmol/L and in the intracellular fluid at a much lower concentration of ∼10 mmol/L. Sodium entry into cells from the extracellular milieu takes place primarily via either of the following: 1. Sodium channels, which can be either voltage-gated or ligand-gated (in many cases neurotransmitter-gated), 2. Sodium transporters, or 3. Sodium pumps. Once sodium ions enter cells, they can act as a critical intracellular second messenger that regulates many cellular functions (Murphy and Eisner, 2009). Furthermore and importantly, the intracellular sodium concentration (10–15 mM) is regarded as a marker for cellular viability.

**FIGURE 6:**
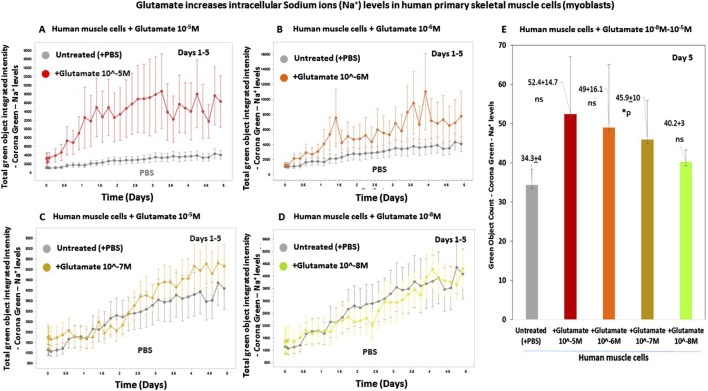
Glutamate increases the levels of intracellular sodium (Na^+^) ions in human primary skeletal muscle cells. **(A–D)** The kinetic graphs show the levels of intracellular sodium (Na^+^) ions in human primary skeletal muscle cells (myoblasts) growing in tissue culture inside an IncuCyte incubator and connected to the IncuCyte system for 5 days. At the onset of the experiment, the muscle cells were fluorescently labeled with CoroNa Green–a fluorescent dye that is a sodium ion (Na^+^) indicator and that exhibits an increase in green fluorescence emission intensity upon binding sodium ions. Then, the cells were either untreated (i.e., added only with PBS for control - gray curves in **(A–D)**, or added with glutamate at a conc. of either 10^−5^M (**(A)**, red curve), 10^−6^M (**(B)**, orange curve), 10^−7^M (**(C)**, yellow curve), or 10^−8^M (**(D)**, green curve). The control group consisted of 12 replicate wells (n = 12), and each of the other experimental groups consisted of six replicates (n = 6). From this point onward, the cells’ CoroNa Green fluorescence (proportional to the level of intracellular sodium ions) was repeatedly measured and analyzed, and the cells were photographed by the IncuCyte technology, every 3 h for days. Note that the Y axis scale is different in Figures **(A–D)** since the IncuCyte software drew each Figure on a different scale that fits the best the data (graphs) shown in that Figure. **(E)** Histograms of the average levels of the CoroNa Green fluorescence (proportional to the level of intracellular sodium ions) in each of the experimental groups, at day 3 of the same experiment. The histogram also shows the averages and SD for each group and statistical values. The statistical analysis comparing glutamate-treated muscle cells to untreated cells yielded the following p values: glutamate 10^-5^M - ns; glutamate 10^-6^M - ns; glutamate 10^-7^M - *p = 0.03, glutamate 10^-7^M - ns. ns = not significant.

For testing if glutamate increases intracellular sodium levels in human skeletal muscle cells, we designed a multistep experimental protocol. Human primary skeletal muscle cells growing in tissue culture were loaded with CoroNa Green. a fluorescent dye that is a highly specific sodium (Na^+^) ion indicator, and that exhibits an increase in green fluorescence emission intensity upon binding sodium ions. Subsequently, glutamate was added at either of four concentrations, 10^−5^M to 10^−8^M to predetermined six replicate wells containing CoroNa Green-labeled skeletal muscle cells. For control, we added only buffer (PBS) to other wells. From that point onward, the skeletal muscle cells were repeatedly measured and analyzed for both their confluence and their CoroNa Green fluorescence, proportional to the level of intracellular sodium ions, and photographed. Finally, the IncuCyte technology yielded graphs of the CoroNa Green fluorescence before and after the addition of glutamate. Figures 6A–D, presenting the CoroNa Green fluorescence curves during 5 days after adding glutamate, and Figure 6E–presenting histograms of the CoroNa Green fluorescence only at day 5 only, as well as the averages, SD, and statistical values of all the experimental groups, show that adding glutamate at either 10^−5^M (Figure 6A; Figure 6E), 10^−6^M (Figures 6B,E), or 10^−7^M (Figures 6C,E) increased significantly the levels of intracellular sodium ions in the human skeletal muscle cells, evident by the elevated levels of CoroNa Green fluorescence. The effect was dose-dependent: the strongest effect was induced by glutamate at 10^−5^M, lower at 10^−6^M, even lower at 10^−7^M, and the lowest at 10^−8^M (Figures 6A–E).

Next, we tested if treatment of the cells with two iGluR agonists, AMPA and NMDA, also increase the intracellular sodium levels in human skeletal muscle cells. In this experiment, we also tested again the effects of glutamate.

**FIGURE 7:**
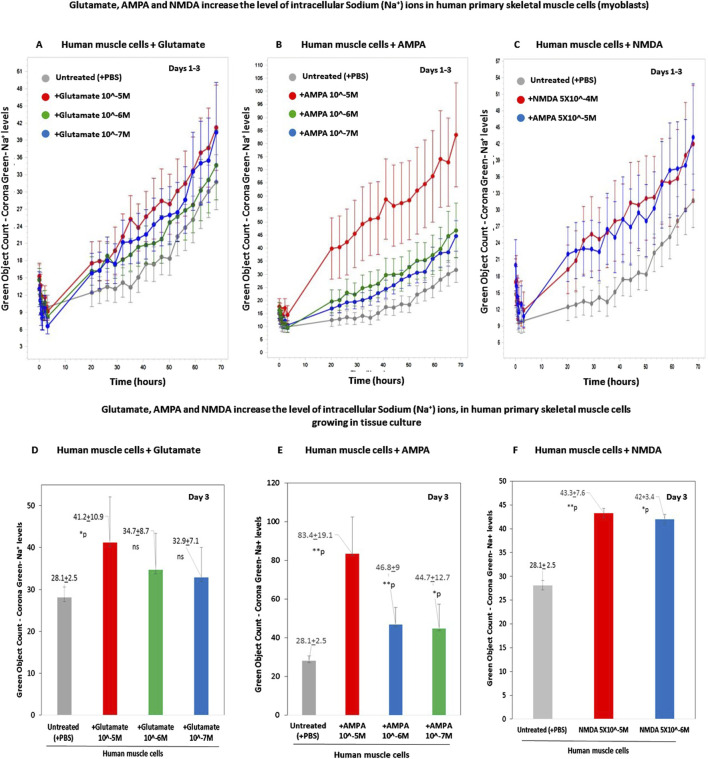
Glutamate, AMPA, and NMDA increase the levels of intracellular sodium (Na^+^) ions in human primary skeletal muscle cells growing in tissue culture. **(A–C)** The kinetic graphs show the levels of intracellular sodium (Na^+^) ions in human primary skeletal muscle cells (myoblasts) growing in tissue culture inside an IncuCyte incubator and connected to the IncuCyte system for 3 days. The skeletal muscle cells were first fluorescently labeled with CoroNa Green–a fluorescent dye that is a sodium ion indicator that exhibits an increase in *green* fluorescence emission intensity upon binding Na^+^ ions. Then, the cells were either untreated (i.e., added only with PBS for control - gray curves in **(A–C)** or added with either glutamate at conc. of 10^−5^M-10^−7^M **(A)**; or AMPA–an iGluR agonist selective to the AMPA receptors - at a conc. of either 10^−5^M or 10^−6^M **(B)** or NMDA - an iGluR agonist selective to the AMPA receptors - at a conc. of 5 × 10^−4^M or 5 × 10^−5^M **(C)**. From this point onward, the cells' CoroNa Green fluorescence (proportional to the level of intracellular sodium ions) was repeatedly measured and analyzed, and the cells were photographed by the IncuCyte technology, every 3 h for 3 days. The control group consisted of 12 replicate wells (n = 12), and each of the other experimental groups consisted of six replicates (n = 6). **(D–F)** Histograms of the average levels of the CoroNa Green fluorescence (proportional to the level of intracellular Sodium ions) in each of the experimental groups, at day 3 of the same experiment. The histogram also shows the averages and SD for each group and statistical values. The statistical analysis comparing either glutamate-treated, AMPA-treated, or NMDA-treated muscle cells to untreated cells yielded the following p values: glutamate 10^-5^M - *p = 0.04; glutamate 10^-6^M - p = 0.14 - ns; glutamate 10^-7^M - p = 0.17 - ns; AMPA 10^-5^M - **p = 0.007; AMPA 10^-6^M - **p = 0.006; AMPA 10^-7^M -*p = 0.02; NMDA 5 × 10^-5^M - *p = 0.05. NMDA 5 × 10^-6^M - **p = 0.01. ns = not significant.

The results are shown in two types of figures: Figures 7A–C, presenting the CoroNa Green fluorescence curves during 3 days, and in Figures 7D–F, presenting histograms of the CoroNa Green fluorescence only at day 3.

In this experiment, we found that glutamate at a concentration of 10^−5^M - 10^−7^M (Figures 7A,D), as well as AMPA at 10^−5^M - 10^−7^M (Figures 7B,E), and NMDA - at either 5 × 10^−4^M or 5 × 10^−5^M (Figures 7C,F), increased the levels of *intracellular s*odium ions in the human primary skeletal muscle cells. It should be noted that all the tested concentrations of both AMPA and NMDA are the well-known active concentrations of these agonists.

**FIGURE 8:**
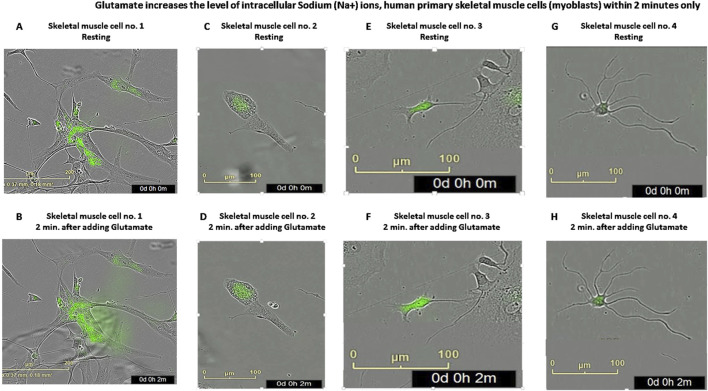
Glutamate increases the level of intracellular sodium (Na^+^) ions in human primary skeletal muscle cells within 2 min **(A–H)** Representative photos of human primary skeletal muscle cells (myoblasts) growing in tissue culture inside an IncuCyte incubator and connected to the IncuCyte system, which were first fluorescently labeled with CoroNa Green–a fluorescent dye that is a sodium ion indicator that exhibits an increase in *green* fluorescence emission intensity upon binding Na^+^ ions and then added with glutamate. Then, the cells were photographed twice, in exactly 2-min interval: first, before any treatment (the upper photos seen in **(A**,**C**,**E**,**G)** - showing untreated CoroNa Green-labeled muscle cells in four different fields of the tissue culture wells) and second - of the same cells exactly 2 min after adding glutamate (the lower photos seen in **(B**,**D**,**F**,**H)**. The comparison of the green areas (i.e., the CoroNa Green fluorescence) before and after adding glutamate in these four representative cells: **(A)** vs. **(B)**; **(C)** vs. **(D)**; **(E)** vs. **(F)** and **(G)** vs. **(H)**, shows the glutamate-induced increase in the intracellular sodium ion level.

Next, we studied if glutamate induces very fast responses in skeletal muscle cells and found that glutamate increased the levels of the intracellular sodium ions within 2 min only (Figure 8), and maybe even sooner in shorter times we could not test.

This finding is shown in the eight representative images of CoroNa Green fluorescence, proportional to the levels of the intracellular sodium ions, inside each of four different human skeletal muscle cells, before and 2 min after adding glutamate (compare in Figure 8: A to B, C to D, E to F, and G to H). The fact that glutamate elevated sodium levels within few minutes (and maybe even less) suggests that glutamate induced these effects via direct activation of GluRs, and that glutamate may contribute to muscle excitation. However, this asssumption requires further investigation and proofs, primarily by electrophysiological recordings.

### 3.6 Autoimmune affinity-purified GluR3B antibodies of epileptic nodding syndrome patients, as well as IgG preparations of these patients that contain the GluR3B antibodies, bind primary human skeletal muscle cells

**FIGURE 9:**
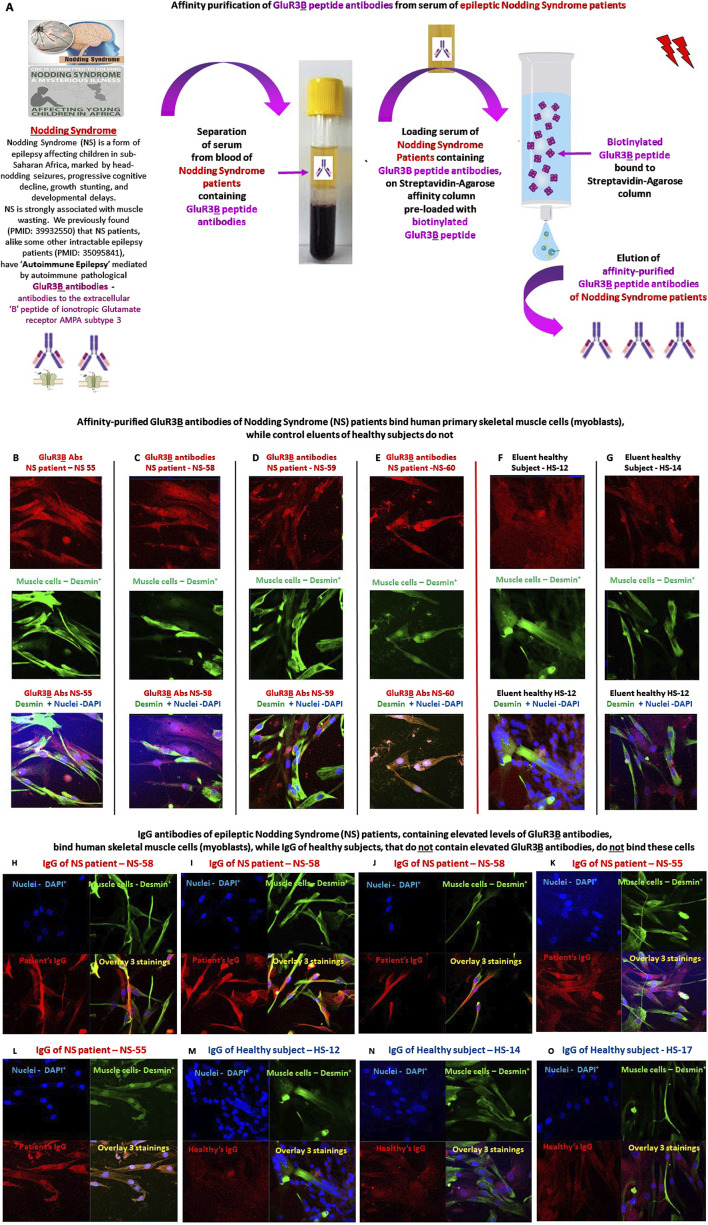
Affinity-purified GluR3B antibodies of Nodding Syndrome (NS) patients and total IgG antibodies of these patients containing the GluR3B antibodies bind primary human skeletal muscle cells **(A)**. Graphical scheme and short explanation of the affinity purification of GluR3 **B** antibodies from Nodding Syndrome (NS) patients, which was performed. The method is also described in the “Methods” and (Levite et al., 2020). **(B–G)** Representative confocal microscopy photos showing that affinity-purified GluR3 **B** antibodies of four South Sudanese NS patients (NS-55 **(B)**, NS-58 **(C)**, NS-59 **(D)**, and NS-60 **(E)**) bind human primary skeletal muscle cells (myoblasts). In contrast, affinity-columns eluent controls of Healthy South Sudanese (HS): HS-12 **(F)** and HS-14 **(G)** do not bind these cells. The upper photos in **(B–G)** show the positive immunostaining of the muscle cells with the NS patients’ IgG GluR3 **B** antibodies (red staining in the upper panels of **(B–E)**) or the negative immunostaining of the healthy subjects’ eluent controls **(F,G)**. The photos in the middle panel show the immunostaining of the same cells with mouse anti-human desmin antibody (seen in green and performed to confirm that the cells are indeed muscle cells.). The photos in the lower panel show overlay of the two antibodies (human GluR3**B** antibodies or controls and anti-desmin antibody) and DAPI. **(H–K)**. Confocal microscopy photos showing that purified IgGs of two representative NS patients: NS-58 (three photos of different cells seen in **(H–J)** and NS-55 (two photos of different cells seen in **(K,L)**), which contain elevated levels of GluR3 **B** antibodies, bind human skeletal muscle cells (myoblasts). In contrast, purified IgGs of three representative healthy subjects: HS-12 **(M)**, HS-14 **(N)** and HS-17 **(O)** do not bind the human skeletal muscle cells. **(J,K)**. For each NS patient or healthy subject in H-0, four photos are shown: staining with DAPI (seen in blue in all the upper left photos), staining with the anti-desmin antibody (seen in green in all the upper right photos), staining with the IgGs of either the patients or the healthy subjects (seen in red in all the lower left photos), and overlays of all the stainings (seen in multicolor in all the lower right photos).

Next, we tested if affinity-purified human autoimmune GluR3 **B** antibodies of few epileptic NS patients bind human skeletal muscle cells, alike the mouse GluR3 **B** mAbs does. The method we used for purification of NS patient’s GluR3 **B** antibodies is drawn schematically in Figure 9A, and described in the “Methods” and in Levite et al. (2020).

We found that the NS patient’s GluR3 **B** antibodies indeed bind the human primary skeletal muscle cells (Figures 9B–E). In contrast, control healthy eluents do not bind the muscle cells (Figures 9F,G).

Next, we isolated all the IgG antibodies from the NS patient’s sera rich in autoimmune GluR3 **B** antibodies, but that also contain many other antibodies, and tested if they bind human skeletal muscle cells. The reason for testing this was that *in vivo*, in the patient’s blood, the GluR3 **B** antibodies are surely present in a heterogeneous milieu of antibodies, not in an isolated form, so if they bind *in vivo* GluR3 expressed on the cell surface of cells, they ought to do so when they are surrounded by many other types of antibodies. We found that the IgGs of the NS patients indeed bind the human skeletal muscle cells (Figures 9H–L). In contrast, control IgGs of healthy subjects do not bind these cells (Figures 9M–O).

### 3.7 Intractable epilepsy patients have elevated levels of GluR3B antibodies in the sera, and their IgGs rich in these GluR3B antibodies bind and affect human skeletal muscle cells

In multiple previous studies on “Autoimmune Epilepsy,” we and others found that some enigmatic intractable epilepsy patients who suffer from very severe and prolonged epilepsy (and very different from NS) have elevated levels of autoimmune GluR3 **B** antibodies in their serum and that these GluR3 **B** antibodies induce multiple pathological effects on the neural cells *in vitro* and *in vivo* and damage the brain (for our most updated comprehensive review on “Autoimmune Epilepsy” GluR3**B** antibodies, which summarizes and cites most of the original studies done by various groups, and for just two recent original studies on the pathological effects *in vitro* and *in vivo* of GluR3**B** antibodies of intractable epilepsy patients and NS patients, the readers are referred to Levite et al. (2020), Levite and Goldberg (2021), Taiwo et al. (2025). These findings indicate that these patients suffer from “Autoimmune Epilepsy.” In some epilepsy patients, it may be their main disease, and in others, it may accompany and contribute substantially to their seizures and other neurological impairments. Some of the intractable epilepsy patients having autoimmune GluR3 **B** antibodies suffer also from motor problems in addition to the seizures, but others do not. So far, the possible binding and harmful effects of epilepsy patients’ GluR3 **B** antibodies or their IgG antibodies that contain high levels of GluR3 **B** antibodies to muscle cells were not tested.

In this specific study focusing primarily of GluR3 in muscle cells and the effects of glutamate on muscles cells, we performed only a very small pilot investigation on antibodies of few intractable epilepsy patients, which differ in many epilepsy characteristics but that all have elevated GluR3 **B** antibodies in serum (as found in our previous studies). The clinical information about the seven epilepsy patients we chose for this small pilot study is shown in Table 1.

**FIGURE 10:**
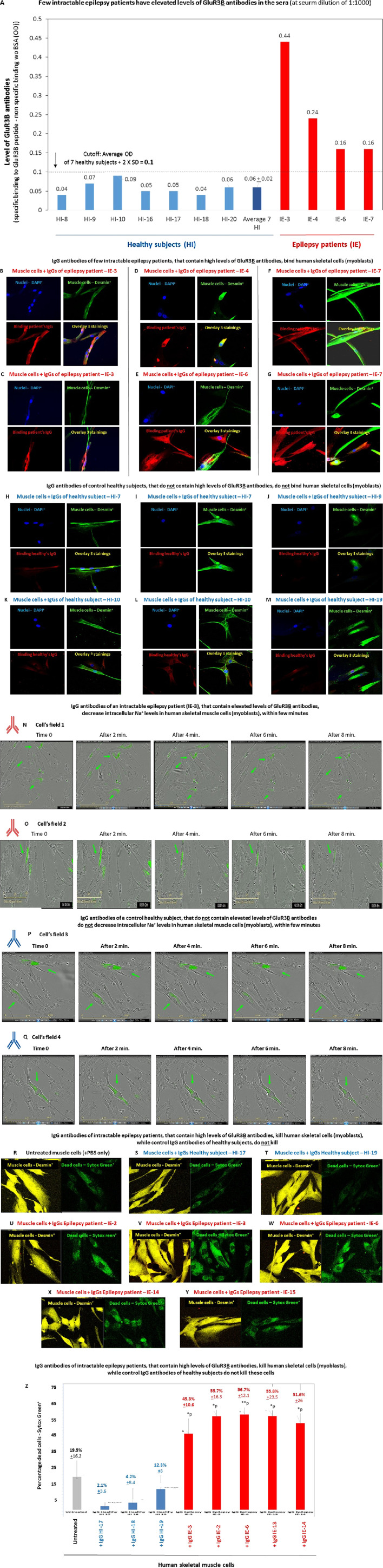
Intractable epilepsy patients have elevated GluR3B antibodies and their IgGs bind and kill human primary skeletal muscle cells **(A)**. Few intractable epilepsy patients have elevated levels of GluR3 **B** antibodies in the sera. The figure shows the results of a representative ELISA, in which we tested for the presence of GluR3 **B** antibodies in the serum of seven healthy subjects, and four of the intractable epilepsy patients whose clinical information is shown in Table 1. Each serum was tested in duplicate ELISA wells, in three serum dilutions: 1:10, 1:100, and 1:1,000) for its parallel binding to either the GluR3 **B** peptide or to control PBS +1% BSA. The figure shows the level of the GluR3 **B** antibodies in very low serum dilution of 1:1,000. The Y axis in this figure shows the value (in OD) referring to the level of the specific GluR3 **B** antibodies was calculated according to the following equation: specific binding (OD) to the GluR3**B** peptide of the antibodies in the given serum in 1:1,000 serum dilution - (minus) the nonspecific binding (OD) of the antibodies in this given serum, in this serum dilution, to the control PBS+ 1% BSA (OD). The experimental cutoff seen in the figure shows the average OD + 2 × SD of the specific binding to GluR3 **B** peptide - the nonspecific binding to the control PBS+ 1% BSA of seven healthy subjects tested in the same 1:1,000 serum dilution, in the same experiment. In this ELISA, the cutoff was 0.1 OD. The figure shows the averages ±SD values of the specific GluR3 **B** antibodies for each healthy subject (blue bars) or epilepsy patients (red bars), as well as the average value of the and healthy subjects and demonstrates that the four epilepsy patients had higher values of GluR3 **B** antibodies than the healthy subjects, above the cutoff. **(B–M)** Confocal microscopy photos showing that purified immunoglobulin G (IgG) antibodies of four representative intractable epilepsy patients: IE-3 (two photos of different cells are seen in **(B,C)**, IE-4 **(D)**, IE-6 **(E)**, and IE-7 (two photos of different cells are seen in **(F,G)**, which contain elevated levels of GluR3 **B** antibodies, bind human skeletal muscle cells (myoblasts). In contrast, purified IgGs of four representative healthy subjects: HI-7 (two photos of different cells are seen in **(H,I)**, HI-9 **(J)**, HI-10 (two photos of different cells are seen in **(K,L)**), and HI-19 **(M)** do not bind these muscle cells. For each intractable epilepsy patients or healthy subjects in **(B–M)**, four photos are shown: staining with DAPI (seen in blue in all the upper left photos), staining with the anti-desmin antibody (seen in green in all the upper right photos), staining with the IgGs of either the patients or the healthy subjects (seen in red in all the lower left photos), and overlays of all the stainings (see in multicolor in all the lower right photos). **(N–Q)**. Representative confocal microscopy photos showing that IgGs of the intractable epilepsy patient (IE-3) (Table 1, (40)), that contain elevated levels of GluR3 **B** antibodies, decrease intracellular Na^+^ levels in Corona green-labeled (myoblasts) within few minutes (**(N,O)**- two cellular fields photographed by the IncuCyte are seen in **(N,O)**). In contrast, IgGs of a control healthy subject do not induce this effect (two cellular fields are seen in **(P,Q)**). In this experiment, the human primary skeletal muscle cells (myoblasts) growing in tissue culture inside an IncuCyte incubator and connected to the IncuCyte system, were first fluorescently labeled with CoroNa Green–a fluorescent dye that is a sodium ion indicator that exhibits an increase in *green* fluorescence emission intensity upon binding Na^+^ ions. Then, the cells were photographed and their CoroNa Green fluorescence analyzed by the IncuCyte. Immediately afterward, and within a maximum of 1 minute, the microtiter plate with the cells was removed from the IncuCyte incubator, the IgGs of the epilepsy patient IE-3 or healthy controls were added very quickly to the appropriate wells, the plate was returned to the incubator, and the cells were photographed again 2, 4, 6, and 8 min after the addition of the IgGs. The photos in **(N,O)** show that the intensity and spread of CoroNa Green fluorescence, i.e., of the intracellular sodium ions, in individual muscle cells decreased within few minutes after addition of the IgG of the epilepsy patient IE-3 (N,O showing two different cellular fields). In contrast, the IgGs of the healthy control did not decrease the CoroNa Green fluorescence **(P,Q)**. **(R–Y)** Representative confocal images of human primary skeletal muscle cells (myoblasts) that grew on slides, showing that IgGs of five intractable epilepsy patients: IE-2 **(U)**, IE-3 **(V)**, IE-6 **(W)**, IE-14 **(X),** and IE-15 **(Y)** kill some of these muscle cells, in comparison to untreated muscle cells **(R)**. In contrast, IgGs of two control healthy subjects: IE-HI **(S)** and HI-19 **(T)**, do not kill these cells muscle cells. In this experiment, live muscle cells were first added with the human IgGs for 1 h and then fluorescently labeled with Sytox green that stains dead cells (seen in green in the right photos in **(R–Y)**), and then with anti-desmin antibody (seen in yellow in the in the left photos in **(R–Y)**). **(Z)** Quantitative analysis of the number of percent of Sytox green^+^ dead skeletal muscle cells in the representative experiments whose confocal microscopy images are seen in **(R–Y)**. Each column shows the average +SD % Sytox green^+^ dead cells, out of all the desmin^+^ cells, counted in five independent cellular fields. The statistical analysis comparing the muscle cells treated with the IgG antibodies of the intractable epilepsy patients to the untreated cells, yielded the following p values: IgG IE-3 - *p = 0.02; IgG IE-2 - **p = 0.01; IgG IE-6 - **p = 0.009; IgG IE-14 - **p = 0.04; IgG IE-13 - *p = 0.02.

Figure 10A presents the results obtained in only one of the ELISAs we performed for detecting GluR3 **B** antibodies in the serum of intractable epilepsy patients and shows that in comparison to nine healthy controls, four epilepsy patients: IE-3, IE-4, IE-6, and IE-7 (Table 1), have elevated levels of GluR3 **B** antibodies in serum. Elevated levels of autoimmune GluR3 **B** antibodies in the other three epilepsy patients: IE-2, IE-13, and IE-14 (data not shown herein) were observed in similar ELISA tests that we performed at other time points and included in previous reports (among them Ganor et al. (2005a), Goldberg-Stern et al. (2014).

Next, since we did not have enough serum from these patients to affinity-purify their specific autoimmune GluR3 **B** antibodies (as we did for the NS patients), we limited ourselves to isolating all their IgGs and testing them. We found that purified IgGs of few of the intractable epilepsy that have high levels of GluR3 **B** antibodies in serum indeed bind human skeletal cells. This is seen in the representative photos of the positive binding of the IgGs of four of these epilepsy patients (Figures 10B–G).

In contrast, the IgGs of four healthy control subjects did not bind the muscle cells (Figures 10H–M).

In the next step of the study, we found out that the purified IgGs of just one intractable epilepsy patient: IE-3* (others not tested), that contain elevated levels of GluR3**B** antibodies, decrease intracellular Na^+^ levels in human skeletal muscle cells, within few minutes (Figures 10N,O). In contrast, the IgGs of healthy subjects do not induce this effect (Figures 10P,Q). *Of note, IE-3 was the only epilepsy patient whose antibodies we tested in this study for their effect on intracellular sodium levels in muscle cells. We chose this specific patient due to two main facts: 1. This patient is in an extremely bad condition (bedridden for the last several years; the patient’s clinical information is summarized in Table 1. In addition, a detailed description of the patient can be found in our recently published article on the multiple *in vitro* and *in vivo* pathogenic effects of the autoimmune antibodies of this patient (Taiwo et al., 2025), 2. We already found and published that IE-3 has elevated IgG antibodies, and three types of glutamate receptor antibodies, directed against AMPA-GluR3 **B**, NMDA-NR1 and NMDA-NR2 peptides, and that the IgG antibodies of IE-3 bind and kill human neural cells *in vitro*, bind neural cells in the hippocampus and cortex and cause neural loss in these brain regions *in vivo* in naïve rats, and most important: induce recurrent generalized tonic–clonic seizures in these naïve rats (Taiwo et al., 2025).

In the last set of experiments in this study, we found that IgGs of few intractable epilepsy patients which have elevated GluR3 **B** antibodies not only bind human skeletal muscle cells, but also kill some of these cells within 1 h only (Figures 10U–W). In contrast, IgG antibodies of healthy subjects do not induce cell death (Figures 10S,T).

In this experiment, cell death was studied by staining the muscle cells with Sytox green–a fluorescent dye that enters only dead/dying necrotic cells. The killing of the muscles cells by the epilepsy patients’ IgG is clearly seen when comparing the representative confocal microscopy images of the Sytox Green^+^ dead cells aside the muscle-specific desmin^+^ cells in the: **A**. untreated muscle cells (Figure 10R), **B**. muscle cells added with IgG of 2 control healthy subjects (Figures 10S,T), **C**. muscle cells added with IgG of 5 intractable epilepsy patients: IE-2 (Figure 10U), IE-3 (Figure 10V), IE-6 (Figure 10W), IE-14 (Figure 10X), and IE-15 (Figure 10Y).

The quantitative graph of the percentage of dead cells (Figure 10Z) shows that the untreated muscle cells had 19.5% ± 16.2 dead cells; the muscle cells added with the IgGs of the three healthy subjects had 2.1% ± 3.6, 4.2% ± 8.4% and 12.3% ± 8 dead cells, respectively; and the muscle cells added with the IgGs of the five epilepsy patients had 45.3% ± 10.6 (*p), 55.7% ± 16.3 (*p), 56.7% ± 12.1 (**p), 55.8% ± 23.5 (*p) and 51.6% + 26 (*p) dead cells, respectively.

These findings suggest that the autoimmune GluR3 **B** antibodies or IgGs of intractable epilepsy patients that contain elevated levels of GluR3 ***B*** antibodies can damage muscle cells and motor function and that their effect is different from that of glutamate itself, and of GluR3 **B** mAb.

## 4 Discussion

All the experiments in this study were performed on a primary culture of human muscle cells (myoblasts), derived from the gastrocnemius of a 32-year-old female following an isolation procedure as described (Amsili et al., 2007). The culture was obtained from the Muscle Tissue Culture Collection (MD-NET, service structure S1, 01GM0302, BMBF, EuroBiobank) at the Friedrich–Bauer Institute. Although only one human muscle cell culture was used in the present scientific investigation, and although all of this study was performed *in vitro*, six main novel findings were discovered. These are discussed below separately and shown schematically in the Graphical Abstract.

The first finding is that human skeletal muscle cells (myoblasts) express on their cell surface functional GluR3, ionotropic glutamate receptor of AMPA type subunit 3. AMPA receptors are composed of four types of subunits GluR1–GluR4 encoded by different genes, which combine to form tetramers. Most AMPA receptors are heterotetrameric, consisting of symmetric “dimer of dimers” of GluR2 and either GluR1, GluR3, or GluR4 (Hansen et al., 2021). The muscle GluR3 found in this study could be either homomeric GluR3 receptor composed only of GluR3 subunits or heteromeric GluR3 receptor composed or the GluR3 subunits assembled with either GluR1 or GluR2. Our study cannot discriminate between these options.

We are not aware of previous findings showing GluR3 RNA expression and cell surface protein expression in human skeletal muscle cells. However, a previous study by Mays et al. found glutamate receptors localize postsynaptically at NMJs of mice and rats (Mays et al., 2009). The researchers used immunostaining with polyclonal antibodies to GluR1 and GluR2/3 subunits of AMPA receptors, and for NMDA NR1 and NR2A subunits of NMDA receptors, and showed their localization to the postsynaptic side of the NMJ in the mouse skeletal muscle fiber.

It is important to note that Personius et al. found that GluRs are expressed at the endplate (the specialized structure at the NMJ where a motor neuron’s axon terminal contacts a muscle fiber) of mice only during neonatal and postnatal development (Personius et al., 2022; Personius et al., 2016). In the present study, we found GluR3 RNA and protein expression, as well as glutamate-induced effects and GluR3**B** antibodies-induced effects on human myoblasts and myotubes growing in tissue culture, not in mature skeletal muscle cells, not in muscle NMJ, and not in mice. Whether GluR3 is expressed also in human mature skeletal muscle cells is still unknown and requires further continuation studies. This information (which is still missing) is important, in order to know and understand: 1. At which age and stage of development GluR3 contributes to the muscle physiological function, 2. At which age and stage in development can glutamate activate GluRs expressed in muscle cells, 3. At which age and stage in development can autoimmune GluR3 **B** antibodies of NS patients and of intractable epilepsy patients bind and damage skeletal muscle cells. Meanwhile, regarding the last point (Gill and Pulido, 2001), we remind readers that NS is a childhood disease that affects previously healthy children with an age of onset typically between 3 and 18 years, and that we found and studied pathological autoimmune GluR3 **B** antibodies in young NS patients. In addition, we found and studied GluR3 **B** antibodies in various young patients with intractable epilepsy (see Table 1).

The second novel finding of this study is that glutamate by itself (i.e., in the absence of any other neurotransmitter or other stimuli/factor) activates human skeletal muscle cells (myoblasts), leading to functional effects. This tentative conclusion is based on the three glutamate-induced effects found in the present study (out of all possible effects that glutamate may induce and not studied yet): 1. Glutamate increased the level of intracellular sodium ions in skeletal muscle cells, lasting for many hours afterwards, 2. Glutamate increased the number of skeletal muscle cells (counted few days later), most probably by inducing cell proliferation, 3. Glutamate increased the skeletal muscle cells’ confluency.

Clearly, these effects are not necessarily mediated through GluR3 or through GluR3 only, and other GluRs could be involved. Be it as it may, the fact that glutamate may have the ability to directly induce proliferation of skeletal muscle cells calls for further investigation on whether injecting glutamate into muscle tissue in humans or animal models will induce muscle cell proliferation *in vivo*. If it will, such injection of glutamate into the muscle may be beneficial and therapeutic for diseases causing muscle loss and functional decline, such as muscular dystrophies, sarcopenia (age-related muscle loss), cancer cachexia, and muscle wasting from other chronic illnesses like heart failure. Other potential applications may include diseases with muscle damage or inflammation, such as chronic musculoskeletal conditions.

The third novel finding of this study is that AMPA and NMDA, two synthetic ionontropic GluR, each by itself, increased the level of intracellular sodium ions in human primary skeletal muscle cells. The finding that both AMPA and NMDA induced these effects, alike glutamate and the GluR3 **B** mAb, suggests the glutamate’s own effects on these cells are mediated by activation of iGluRs, rather than by a metabolic mechanism.

The fourth novel finding of this study is that mouse GluR **B** mAb, directed against the extracellular GluR3 **B** peptide of GluR3, binds human skeletal muscle cells and eventually leads to an increase in the muscle cells’ number. We assume, but did not prove directly, that the GluR3 **B** mAb alike glutamate itself, induced this effect by inducing *de novo* proliferation of the skeletal muscle cells. We further assume that the GluR3 **B** mAb induced this effect by direct activation of GluR3 in the muscle cells. This assumption is based on three previous studies and publications. First, we previously showed that GluR3 **B** antibodies raised in mice by immunization with the GluR3 **B** peptide, activated *in vitro* the AMPA receptor ion channel in neurons and induced the characteristic ion currents (Levite et al., 1999). Second, we showed also that affinity-purified GluR3 **B** autoantibodies activate both homomeric GluR3 receptors and heteromeric AMPA receptor channels complexes composed of GluR3(o)/GluR2(o) or GluR3(o)/GluR2(i), without the requirement of neuronal, glial or blood ancillary molecules (Cohen-Kashi Malina et al., 2006). *In vivo*, AMPA receptors are present mostly as heteromeric complexes (Cohen-Kashi Malina et al., 2006).

Third, GluR3 and GluR3**B** antibodies have been shown to activate a subset of AMPA receptors and revealed an agonist binding site in the extracellular “B” peptide/region of the receptor (Twyman et al., 1995) (Carlson et al., 1997).

Based on the findings of the present paper regarding the binding and effects of GluR3 **B** mAb on human skeletal muscle cells, we raise an hypothesis which calls for further investigation, of a potential therapeutic use of GluR3**B** mAb for inducing renewal, growth and proliferation of skeletal muscles when needed (Samia et al., 2022; Idro et al., 2024).

The fifth novel finding of this study is that human autoimmune GluR3 **B** antibodies of few epileptic NS patients bind human skeletal muscle cells. A preparation of all the IgG antibodies present in the blood of these patients, rich in GluR3 **B** antibodies, also bind human skeletal muscle cells.

It is very important to remember that NS is strongly associated with muscle wasting and low muscle mass and can involve a temporary loss of neck muscle tone (Abd-Elfarag et al., 2021; Samia et al., 2022). The NS-related finding revealed in the present study showing that NS patients’ autoimmune GluR3 **B** antibodies can bind human primary skeletal muscle cells, leads us to hypothesize that these antibodies may do so *in vivo* as well, and consequently damage the human muscle cells and impair motor function, alike they bind, activate, and kill human neural cells and cause brain damage, behavioral impairments, and epilepsy (all these effects are summarized and discussed in Levite et al. (2021) and by doing so, we contribute to the NS patient’s repeated nodding, muscle wasting, and other motor problems.

The sixth novel finding of this study is that IgGs of few intractable epilepsy patients (very different from NS), that are rich in autoimmune and GluR3**B** antibodies that we already found to bind and kill neural cells *in vitro* and *in vivo*, seem to have the ability also to bind and damage GluR3-expressing human skeletal muscle cells.

If GluR3 is expressed in the patients’ own muscle cells (not shown yet), and if the autoimmune GluR3 **B** antibodies bind these cells, they may damage them and impair the patients’ motor function.

Taken together, our novel findings stimulate subsequent investigations on multiple physiological, pathological, and pharmacological topics, as listed below. 1.GluR3 may have an important physiological role in muscle cell activation, function, proliferation, survival, and communication with other cells; 2. Possible GluR3 defects and impaired GluR3 function (due to either genetic, epigenetic, autoimmune, infectious, inflammatory, or other factors) could lead to impaired muscle cell activation, function, proliferation, survival, and communication with other cells; 3. Glutamate, at physiological concentration, and by direct activation of GluR3 and/or other GluRs expressed in skeletal muscle cells, may affect beneficially muscle cell survival, growth, and function, 4. Glutamate, iGluR agonists, and/or GluR3 **B** mAb may have beneficial and therapeutic effects for muscle disease-, injury-, and age-related sarcopenia, 5.Autoimmune GluR3 **B** antibodies of NS patients and/or other epilepsy patients may bind GluR3 in skeletal muscle cells, damage these cells, and induce muscle dysfunction and motor problems. The importance of follow-up studies along these directions and hypotheses is self-evident from several solid facts. First, muscle damage or disease leads to progressive weakness and disability, manifesting in more than 100 different human disorders. According to the World Health Organization (WHO), approximately 1.71 billion people have musculoskeletal conditions worldwide. Musculoskeletal impairments significantly limit mobility and dexterity, leading to early retirement from work, lower levels of wellbeing, and reduced ability to participate in society. Moreover, the number of people living with musculoskeletal conditions and associated functional limitations is growing because of population growth and aging.

Second, glutamate is reduced in several muscle diseases, and few disease-associated factors, e.g., hypoxia and oxidative stress, were found to be involved in the disturbed glutamate metabolism (Rutten et al., 2005). In addition, neuromuscular glutamate receptors have been shown to impact reinnervation following nerve crush (Personius et al., 2022; Personius et al., 2016).Third, a significant proportion of enigmatic intractable epilepsy patients have pathological autoimmune GluR3 antibodies (Levite and Goldberg, 2021).

## Data Availability

The original contributions presented in the study are included in the article, further inquiries can be directed to the corresponding author.
